# Contextual influences across the cancer control continuum

**DOI:** 10.1093/jncics/pkac089

**Published:** 2023-01-10

**Authors:** Geetanjali D Datta, Hazel B Nichols

**Affiliations:** Department of Medicine, Research Center for Health Equity, Cedars-Sinai Medical Center, Los Angeles, CA, USA; Department of Epidemiology, University of North Carolina Gillings School of Global Public Health and Lineberger Comprehensive Cancer Center, Chapel Hill, NC, USA

In this issue of the Journal, Ho and colleagues (1) use data from the Surveillance, Epidemiology, and End Results database to demonstrate associations between county-level income, socioeconomic status (SES), and urban–rural status and cardiovascular disease (CVD) mortality among breast cancer survivors. It is a valuable contribution to the expanding focus on the role of social determinants of health to mortality risk among cancer survivors.

Standardized mortality ratios (SMRs) help us compare mortality rates in a population of interest, in this case breast cancer survivors, with the general population. The standardization occurs on characteristics for which we possess population-level mortality data—often the year, age and sex distribution, and racial group distribution for an area. In public health disciplines, race is widely recognized as a social construct that embodies the United States’ long history of human classification systems that reinforce existing structures of hierarchy and power. With this recognition, it warrants consideration of what, in fact, we are standardizing on when we standardize on race (2,3). When we calculate the expected number of deaths within an area, given its racial composition, and we observe a similar number of deaths (or an SMR close to 1), we may inadvertently disguise excess mortality due to social conditions that are patterned by structural racism. We may want to exercise caution that standardization on race does not unintentionally result in the underestimation of associations with measures of advantage by attributing some of their variation to the racial composition of an area.

The patterns of relative risks observed are intuitive—breast cancer survivors in the highest income and SES groups, and the most urban, had better CVD mortality profiles than those in the lowest income or SES, or most rural, groups. The authors highlight meaningful potential contributors to the higher observed CVD mortality, including lower access to cardiovascular screening at the time of treatment decision making, less frequent use of precision medicine–informed treatment modalities, and travel-related barriers to specialized cardio-oncology care. It may also be worth noting the patterns of SMRs. Overall, CVD mortality is the same, or lower, for breast cancer survivors compared with the general female population in all but the very lowest SES and income categories. When stratified according to localized vs regional stage breast cancer, this pattern deviates somewhat. Women with localized breast cancer have SMRs that indicate CVD mortality rates that are lower, or the same, as the general population across county-level income, SES, and rurality categories. Women with regional breast cancer, however, typically had higher CVD mortality than the general population in more rural settings and in lower SES and lower income counties. One explanation for this pattern could be that women with sufficient health-care access to engage in breast cancer screening form a higher proportion of those with earlier-stage disease, and that same health-care access is similarly beneficial for CVD mortality risk.

The authors recommend future studies address the intersection of upstream social conditions and area effects in relation to long-term cancer survivorship outcomes. Theoretical frameworks and approaches can be useful tools to motivate geographic-based analyses and guide hypothesis generation even if the goal is descriptive (4). Although as cancer epidemiologists and outcomes researchers we are generally trained to examine proximal causes as levers for intervention, the contextual influences on health, such as SES, can lend themselves well to describing or explicating more distal influences. For example, one useful way to frame these types of analyses and their interpretation is the fundamental cause theory. This theory, presented by Link and Phelan in 1995 (2), proposes that social causes, such as SES, influence disease because access to resources influences one’s ability to minimize exposure to risk and mitigate disease sequalae once it has occurred. Social conditions are “fundamental” causes of disease that remain even if downstream mechanisms change. With this perspective we can start to conceptualize a broader set of potential influences and interventions.

Because residential location offers opportunities and constraints that affect health via multiple sectors, the Health in All Policies (HiAP) approach can be useful in explaining or generating hypotheses about observed patterns. HiAP is a framework that focuses on the role of multiple public sectors, including housing, education, and environment, in generating health (5,6). Because public policies can be influenced by structural bias (7,8), HiAP also has a strong equity lens. In the case of cardiovascular outcomes, social policy–related influences may include the availability of infrastructure permitting physical activity (such as parks and bike lanes), safety (e.g., from crime or automobile collisions), and the availability of fresh, nonprocessed foods (which may not be available in food deserts), as noted by the authors. In addition to behavioral influences on health, residential location can influence cancer and cardiovascular-related outcomes as the opportunities and constraints offered by geographic location can cause biologic changes through the concept of embodiment, the process by which social conditions manifest in our biology (9). These biologic changes can influence multiple disease processes at once, including cancer and CVD.

Using the perspectives above might suggest future research should investigate policy-relevant area units. The current study used the smallest available data units, which were county-level measures. However, counties have variable policy relevance across the country. There is also the potential for a great deal of heterogeneity within counties themselves. In the current study, someone who resides in Bel Air, an area of Los Angeles County where the median income is greater than $200 000/y, would be assigned the same SES value as someone who resides downtown, where median income is approximately $15 000/y (10). Research to directly inform policy would benefit from exploration of these patterns at both larger (states) and smaller (municipalities) geographic units to inform administrative decisions.

We are all influenced by our environments; thus, we encourage the continued investigation of contextual influences across the cancer control continuum. Integration of theory can aid in structuring analyses, interpreting results, and generating hypotheses for further research. We have presented just a few brief example approaches here. Many more theories, approaches, and frameworks [including ecosocial theory (11), society to cells (12), structural racism (7), etc.] have been used in cancer-related research and health research more generally. A focus on contextual contributors across the cancer control continuum both broadens our understanding and expands the opportunities for meaningful intervention to improve cancer and CVD outcomes and health in general.

## Data Availability

No original data were used for this work.

## Notes

**Role of the funder:** Not applicable.

**Disclosures:** The authors declare they have no conflicts of interest.

**Author contributions:** Geetanjali D. Datta (Conceptualization, Writing – original draft, Writing – review & editing), Hazel B. Nichols (Conceptualization, Writing – original draft, Writing – review & editing).
